# An Exploratory Study on the Relationship Between Idiopathic Epilepsy and Sleep in Dogs

**DOI:** 10.1111/jvim.70026

**Published:** 2025-03-15

**Authors:** Alejandra Mondino, Julie Nettifee, Karen R. Muñana

**Affiliations:** ^1^ Department of Clinical Sciences North Carolina State University Raleigh North Carolina USA

**Keywords:** antiepileptic drugs, epilepsy‐related sleep disturbances, seizures, sleep–wake cycle, translational research

## Abstract

**Background:**

The bidirectional relationship between epilepsy and sleep is well characterized in humans but has not been thoroughly explored in dogs.

**Objective:**

Evaluate sleep differences between dogs with idiopathic epilepsy and healthy controls and determine associations among epilepsy characteristics, antiseizure medications (ASMs), and sleep.

**Animals:**

Sixty‐four dogs with idiopathic epilepsy and 64 non‐epileptic control dogs matched by age, breed, and sex.

**Methods:**

Owners of all dogs completed an online survey that included questions regarding signalment, epilepsy history if applicable, and sleep. Owners of epileptic dogs were asked whether their dogs experienced changes in sleep after a seizure. Sleep scores were calculated using the Sleep and Nighttime Restlessness Evaluation (SNoRE) questionnaire, consisting of two factors: “sleep quality” and “sleep interruptions caused by dreaming.” Data were analyzed for differences in sleep scores between epileptic and control dogs and any effects of seizure frequency, cluster seizures, and ASMs.

**Results:**

Significant differences in sleep scores were identified between epileptic dogs and controls (median, 12 vs. 8, respectively; *p* < 0.001), driven by poorer sleep quality in the epileptic group. No significant associations were found between sleep scores and seizure frequency, clusters, or ASM use. Fifty‐nine percent of dog owners noticed changes in sleep patterns after a seizure, most often increased sleep duration.

**Conclusions and Clinical Importance:**

Dogs with idiopathic epilepsy experience negative changes in sleep, emphasizing the importance of monitoring and managing sleep disturbances in this cohort. Larger, multicenter studies controlling for confounding factors are needed to better understand the impact of epilepsy on sleep.

**Abbreviations**
ASMs: antiseizure medications
CBD: cannabidiol
IQR: interquartile range
IVETF: International Veterinary Epilepsy Task Force
KBr: potassium bromide
MCT: medium‐chain triglycerides
NREM: non‐REM sleep
REM: rapid eye movement sleep
SNoRE: Sleep and Nighttime Restlessness Evaluation

## 1 Introduction

Epilepsy is one of the most common neurological disorders in dogs, affecting 0.62%–0.82% of all dogs in first opinion practices [1, 2, 3], with a much higher prevalence in predisposed breeds such as border collies [1], Labrador retrievers [4], Belgian shepherds [5], and Petit Basset Griffon Vendéens [6], among others [7]. In humans, the complex interaction between sleep and epilepsy has been well documented since ancient times. Aristotle noted the predominance of seizures during sleep [8], which was later substantiated by research showing increased seizure frequency and interictal epileptiform discharges during non‐rapid eye movement (NREM) sleep [9, 10]. This finding is thought to be caused by the increase in neuronal synchronization [11].

Humans with epilepsy are two to three times more likely than the general population to suffer from sleep disorders such as insomnia, restless leg syndrome, and rapid eye movement (REM) sleep disorders [12, 13]. This situation can result in a vicious cycle because sleep deprivation is a precipitating factor for seizures [14]. Antiseizure medications (ASMs) further influence this dynamic, with some drugs, such as phenytoin and phenobarbital, negatively affecting sleep quality and quantity [15]. Newer ASMs, on the other hand, have shown little negative effect or even improvement of sleep; gabapentin, for example, increases sleep duration and depth [16]. Conflicting results have been reported with levetiracetam, with some studies showing increased sleep efficiency and sleep time [17, 18], whereas others report decreased sleep time and increased daytime sleepiness [19], or identify no effect [20].

Dogs, like humans, can suffer from sleep disorders such as narcolepsy, REM sleep disorder, and age‐related sleep impairment [21, 22]. Notably, potassium bromide (KBr), an ASM used in dogs, has been shown to be efficacious in the treatment of REM sleep disorder [23]. However, the relationship between sleep and epilepsy in dogs remains largely unexplored. A recent study used activity monitors to indirectly assess sleep and activity levels in epileptic dogs treated with at least one ASM [24]. Although the investigators found decreased overall activity in epileptic dogs compared with that of non‐epileptic controls, no significant differences in sleep scores were detected. Reliance on averaged activity data over long periods of time likely resulted in missed time‐specific changes. We aimed to further evaluate sleep patterns in epileptic dogs compared with those in healthy controls. By utilizing a recently validated sleep questionnaire designed specifically for dogs [25], we sought to determine differences in sleep between dogs with idiopathic epilepsy and healthy controls. Additionally, we examined how variables such as seizure frequency, cluster seizures, and ASM administration influenced sleep in these dogs.

## 2 Materials and Methods

We investigated sleep patterns in dogs with idiopathic epilepsy compared with healthy control dogs using a validated sleep questionnaire. The study was approved by the NC State University Institutional Animal Care and Use Committee (IACUC) under protocol number 23–334.

### 2.1 Dogs

Epileptic dogs were recruited from a database maintained by the Companion Animal Epilepsy Laboratory at NC State University, through which owners voluntarily provide information on their dogs if they are interested in participating in clinical studies on epilepsy. Inclusion criteria required dogs to have a confirmed diagnosis of idiopathic epilepsy, meeting Tier II confidence level according to International Veterinary Epilepsy Task Force (IVETF) guidelines, including normal laboratory test results and normal brain magnetic resonance imaging and cerebrospinal fluid analysis [26]. Owners of eligible dogs were sent an email invitation with a survey link. Control dogs were recruited by advertisements via the NC State University College of Veterinary Medicine and social media platforms that included a survey link. Control dogs included in the study were matched to epileptic dogs based on sex, age (< 1 year, 1–5 years, or > 5 years of age), and American Kennel Club breed group (herding, hound, toy, sporting, non‐sporting, terrier, or working). Dogs with a history of seizures or any reported movement disorder were excluded. For the control group, the first dog identified from the list of eligible dogs that was a match for an epileptic dog was selected for inclusion.

Two distinct questionnaires were developed for the study using a commercially available survey platform (Qualtrics XM, Qualtrics International Inc., Provo, UT). The epileptic dog questionnaire (File S1) included sections on signalment (date of birth, breed, sex) and epilepsy history (age at first seizure, diagnostic tests performed, seizure frequency categorized as daily, weekly, monthly, every 2–6 months, or yearly, presence of cluster seizures, and ASMs being administered). An additional question asked whether the dog exhibited changes in sleep or wakefulness after a seizure with predefined responses: “Yes, they sleep more than usual,” “Yes, they have difficulty falling asleep,” or “Other,” with a free‐text option for further description and details on whether the dog received other medications or supplements. Owners also completed the Sleep and Nighttime Restlessness Evaluation (SNoRE) questionnaire [25], which consists of six questions grouped into two factors. Factor 1 (sleep quality) assesses the dog's ability to fall asleep at bedtime, sleep continuously, the need to urinate or defecate during the night, and breathing interruptions during sleep. Factor 2 (sleep interruptions caused by dreaming) includes two questions evaluating the extent to which vocalizations and twitching wake the dog up. Each question evaluates the dog's sleep over the past 7 days, using a scale from 1 to 10, where higher scores reflect poorer sleep. The total SNoRE score can range from 6 to 60.

The control dog questionnaire included similar sections but excluded epilepsy history (File S2). Control dog owners were asked about signalment, previous and current medical conditions, any medications or supplements, and seizure history. They also completed the SNoRE questionnaire, identical to the one used for epileptic dogs. No questions regarding diet were included in either questionnaire.

### 2.2 Statistical Analysis

Data were evaluated for normality using the Shapiro–Wilk test. Normally distributed data are presented as mean ± SD, whereas non‐normally distributed data are presented as median and interquartile range (IQR). Regarding the SNoRE questionnaire, differences between epileptic and control dogs were calculated for the total score, as well as for factor 1 and factor 2 separately using the Wilcoxon signed‐rank test. Differences in the total SNoRE score as well as each individual factor also were calculated for dogs based on seizure frequency, presence or absence of cluster seizures, number of ASMs (grouped as 0, 1–2, or > 2), and specific ASMs administered. Differences between groups were analyzed using the Wilcoxon signed‐rank test (paired data) or Kruskal–Wallis test (to compare three or more groups). In analyses with multiple comparisons, *p*‐values were adjusted using the Benjamini–Hochberg method to control the false discovery rate at 0.05. All statistical analyses were performed using commercially available software (JMP Pro 16, JMP Statistical Discovery LLC, Cary, NC), with significance established at *p* ≤ 0.05.

## 3 Results

### 3.1 Population

Seventy‐four questionnaire responses were obtained for epileptic dogs. Eight were excluded because of partial answers and two for being repeated for the same dog. In those cases, the first response was utilized. This resulted in a final sample of 64 dogs included in the study. The median age was 5.6 years (IQR, 3.12–7.5 years). Twenty‐two were female (21 spayed) and 42 were male (36 neutered). For the control dogs, 548 responses were received. Twelve were excluded based on a history of seizures and an additional 53 were excluded for providing incomplete answers to the questionnaire. The first 64 controls that matched the epileptic dogs based on age, breed group, and sex were selected for inclusion in the study. Twenty‐two of these controls were female (20 spayed) and 42 were male (35 neutered). The median age was 5.4 years (IQR, 2.8–8.5 years). No significant differences in sex (*p* = 1.00) or age (*p* = 0.90) were found between the epileptic and control groups.

The breed distribution for both groups included 17 mixed breed dogs, 15 herding dogs, 11 working dogs, 10 sporting dogs, 4 toy dogs, 3 hound dogs, 2 non‐sporting dogs, and 2 terrier dogs. In the epileptic group, the most common breeds were border collie (*n* = 6), Australian shepherd (*n* = 5), and Saint Bernard (*n* = 3). Additionally, there were two dogs each of the following breeds: American pitbull terrier, boxer, chihuahua, Cavalier King Charles spaniel, French bulldog, German shorthaired pointer, Great Dane, and Shetland sheepdog. Breeds represented once included clumber spaniel, English springer spaniel, golden retriever, Greater Swiss Mountain dog, German shepherd, Siberian husky, Irish setter, Italian greyhound, labrador retriever, Lagotto Romagnolo, Portuguese water dog, perro de presa canario, rottweiler, and wirehaired pointing griffon. Data regarding the use of additional supplements or medications were collected from owners of 40 dogs; an error in the questionnaire did not allow all owners to complete this question. Of these, 13 dogs (32.5%) were not on any supplements or medications other than ASMs. Twenty‐five dogs (62.5%) were given supplements, with most of them receiving more than one. Specifically, nine were on cannabidiol (CBD) oil, seven were on medium‐chain triglyceride (MCT) oil, and four were on a commercial diet supplemented with MCT oil. Other supplements included probiotics (five dogs), glucosamine (four dogs), milk thistle (three dogs), melatonin (three dogs), fish oil (three dogs), S‐adenosylmethionine combined with silybin (two dogs), an unspecified mushroom blend (two dogs), a muscle supplement (one dog), and a multivitamin supplement (one dog). Additionally, four dogs (10%) were receiving additional medications, with two on telmisartan, one on levothyroxine, and one on grapiprant.

In the control group, the most represented breeds were Australian shepherds (*n* = 7), border collies (*n* = 3), and golden retrievers (*n* = 3). There were two dogs of each of the following breeds: boxer, chihuahua, dachshund, English labrador, French bulldog, labrador retriever, Siberian husky, and wirehaired pointing griffon. Breeds represented once included American pitbull terrier, American Staffordshire terrier, Belgian Malinois, Belgian Tervuren, Bernese mountain dog, Cavalier King Charles spaniel, cocker spaniel, Dutch shepherd, Great Dane, Greater Swiss mountain dog, greyhound, mastiff, old time Scotch collie, Portuguese water dog, perro de presa canario, rottweiler, and toy poodle. Of these dogs, 49 (76.5%) did not have any current medical condition. Of the remaining 13 dogs (23.5%), 3 had anxiety, 3 had arthritis, 3 had allergies, 2 had luxating patellas, and 1 each had bilateral retinal detachment and focal intestinal lipogranulomatous lymphangitis.

Forty‐two dogs (65.6%) were not on any medications or supplements. Of the 22 dogs (34.4%) receiving treatment, 9 (41%) were on supplements, 10 (45.4%) were on medications, and 3 (13.6%) received both medication and supplements. Some dogs received multiple medications or supplements concurrently. Specifically, of the dogs on supplements, three were receiving probiotics, three were receiving glucosamine, three were receiving fish oil, one was on an unspecified skin supplement, one was on an anal gland supplement, one was on muscle supplements, and one was on mushroom powder. Among those dogs that were receiving medications, four were on oclacitinib, three were on trazodone, two were on fluoxetine, two were receiving anti‐nerve growth factor monoclonal antibody injections, and one was on prednisone.

### 3.2 Characterization of Epileptic Dogs

Thirty (46.9%) of the epileptic dogs had seizures that were reported to occur monthly, 13 dogs (20.3%) had a weekly seizure frequency, 11 dogs (17.2%) had one seizure every 2–6 months, 9 dogs (14.1%) had a seizure approximately every year, and 1 dog (1.6%) had seizures daily. For analysis, and because of the small number of animals in certain categories, seizure frequency groups were consolidated into three categories: daily or weekly, monthly, and less frequent than monthly. Fifty‐two of the dogs (81.3%) had experienced cluster seizures, whereas 12 (18.7%) only had isolated seizures. Most of the dogs had postictal behavior noticed by their owners (*n* = 46, 71.9%).

Fifty‐eight dogs (90.6%) were receiving ASMs. The number of ASMs administered were 1 in 4 dogs, 2 in 19 dogs, 3 in 22 dogs, 4 in 12 dogs, and 5 in 1 dog. The most commonly used ASMs were phenobarbital (*n* = 51), levetiracetam (*n* = 49), zonisamide (*n* = 28), and KBr (*n* = 21). Other maintenance ASMs included gabapentin (*n* = 1), pregabalin (*n* = 4), and topiramate (*n* = 1). Because of the small number of dogs on these latter drugs, we did not analyze differences in sleep scores associated with them.

When asked whether the owners had noted changes in their dogs' sleep or wakefulness after a seizure, 44 (68.8%) responded “Yes,” 19 (29.7%) responded “No,” and one (1.6%) did not answer. Among those reporting changes, 25 (59.5%) noted their dogs slept more than usual, 8 (19%) reported difficulty falling asleep, and 8 (19%) chose the “other” option. The remaining three owners selected multiple options; one reported both increased sleep and difficulty falling asleep, another indicated both increased sleep and “other,” and a third noted difficulty falling asleep along with “other.” Descriptions in the “other” category included dogs trying to sleep but being awakened by episodes, sleeping with eyes open, having highly variable sleep patterns, and taking about an hour to settle before sleeping normally.

### 3.3 Sleep Differences Between Epileptic Dogs and Controls

**FIGURE 1:**
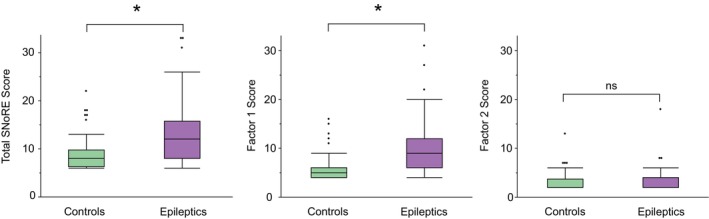
Differences in SNoRE scores between control dogs (green) and dogs with epilepsy (purple). Dogs with epilepsy exhibited significantly higher total scores and factor 1 scores, with no differences observed in factor 2. Box plot shows the median (center line), with the box representing the interquartile range (IQR). Whiskers extend 1.5 times the IQR from the box edges, and the dots represent outliers. Asterisk indicates significant differences. ns: non‐significant.

Significant differences in sleep questionnaire scores were identified between the epileptic and control groups (Figure 1). For the full questionnaire, epileptic dogs had a median score of 12 (IQR, 8–15.7; range, 6–33), whereas control dogs had a median score of 8 (IQR, 6.2–9.7; range, 6–22; *p* < 0.001). This difference was mainly driven by factor 1 (sleep quality). For this factor, epileptic dogs had a median score of 9 (IQR, 6–12; range, 4–31), whereas the control dogs' median score was 5 (IQR, 4–6; range, 4–16; *p* = < 0.001). No significant differences were observed for factor 2 (sleep interruptions caused by dreaming). Epileptic dogs had a median score of 2 (IQR, 2–4; range, 2–18), and control dogs also had a median score of 2 (IQR, 2–3.7; range, 2–13; *p* = 0.99).

### 3.4 Associations Among Seizure Characteristics, ASMs, and Sleep

We found no significant association between SNoRE scores and seizure frequency, the presence of cluster seizures, or the number of ASMs administered (Table 1). Additionally, none of the ASMs evaluated had a substantial effect on sleep scores.

**TABLE 1:** Relationship between SNoRE scores and variables such as seizure frequency, presence of cluster seizures, and ASMs administered.

	Total SNoRE score	Factor 1 score	Factor 2 score
Seizure frequency	*p* = 0.73/adj *p* = 0.93	*p* = 0.49/adj *p* = 0.95	*p* = 0.07/adj *p* = 0.24
Daily/weekly (*n* = 14)	12.5 (7.7–26.5)	9.5 (5.7–16.7)	2.5 (2–5.2)
5 Monthly (*n* = 30)	12 (8–17.2)	9.5 (6–14.2)	2 (2–2)
> once a month (*n* = 20)	12 (8.2–13.7)	7 (6–10)	2 (2–2)
Cluster seizures	*p* = 0.22/adj *p* = 0.93	*p* = 0.54/adj *p* = 0.95	*p* = 0.05*/ adj *p* = 0.24
Yes (*n* = 52)	12 (8–15.7)	8 (6–12.7)	2 (2–3)
No (*n* = 12)	13 (11.5–17.7)	9.5 (7–11.7)	4 (2–5.5)
Number of ASMs	*p* = 0.77/adj *p* = 0.93	*p* = 0.73/adj *p* = 0.95	*p* = 0.40/adj *p* = 0.54
0 (*n* = 6)	13 (7.5–15.5)	8.5 (5.5–11.2)	3 (2–6.5)
1–2 (*n* = 23)	12 (10–16)	9 (7–12)	2 (2–4)
> 2 (*n* = 35)	12 (8–17)	9 (6–13)	2 (2–4)
Phenobarbital	*p* = 0.48/adj *p* = 0.93	*p* = 0.91/adj *p* = 0.95	*p* = 0.17/adj *p* = 0.30
Yes (*n* = 51)	12 (8–15)	9 (6–12)	2 (2–4)
No (*n* = 13)	13 (8.5–19.5)	8 (6.5–14.5)	2 (2–6)
Levetiracetam	*p* = 0.89/adj *p* = 0.93	*p* = 0.74/adj *p* = 0.95	*p* = 0.98/adj *p* = 0.98
Yes (*n* = 49)	12 (8–17)	9 (6–13)	2 (2–4)
No (*n* = 15)	13 (8–15)	9 (6–12)	2 (2–4)
Zonisamide	*p* = 0.93/adj *p* = 0.93	*p* = 0.95/adj *p* = 0.95	*p* = 0.46//adj *p* = 0.54
Yes (*n* = 28)	12 (8–17.7)	8.5 (6–14.5)	2 (2–3.7)
No (*n* = 36)	12 (8.5–14)	9 (6–11.7)	2 (2–4)
KBr	*p* = 0.89/adj *p* = 0.93	*p* = 0.74/adj *p* = 0.95	*p* = 0.17//adj *p* = 0.30
Yes (*n* = 21)	12 (8.5–16)	10 (6–14)	2 (2–2.5)
No (*n* = 43)	12 (8–16)	8 (6–12)	2 (2–4)

*Note:* Data are presented as median (interquartile range, IQR). For this analysis, *p*‐values were adjusted for multiple comparisons using the Benjamini–Hochberg method to control the false discovery rate at 0.05. Both original and adjusted *p*‐values (adj *p*) are shown in the table.

Abbreviations: ASMs, antiseizure medications; KBr, potassium bromide.

## 4 Discussion

We found that, similar to humans, dogs with epilepsy experience negative changes in sleep. To evaluate these changes, we employed the SNoRE questionnaire, which recently was developed to study sleep in dogs and validated using concurrent evaluation with polysomnography and activity monitoring [25]. The median SNoRE score for epileptic dogs was 50% higher than that of control dogs. This overall difference in SNoRE scores was mainly attributed to factor 1, whereas no significant differences were found in factor 2. Factor 1 assesses key elements of sleep quality, including the ability to fall asleep at bedtime, ability to sleep continuously through the night, nighttime urination or defecation, and breathing interruptions. In contrast, factor 2 focuses specifically on REM sleep disturbances. As expected, the primary difference between epileptic and non‐epileptic dogs was in sleep quality, consistent with findings in humans [12, 13, 27]. However, in elderly people, epilepsy has been associated with REM sleep behavior disorder in 12.5% of cases [28]. Given the low prevalence of REM sleep disorder in dogs, future studies should investigate whether a similar association exists between epilepsy and REM sleep disorder in a larger population of dogs.

Our findings differ from those of a recent study that did not find differences in sleep scores between dogs with and without epilepsy [24]. This study relied on activity monitors for sleep assessment, which serve as an indirect measure of sleep. Although these monitors can yield valuable information about the overall activity levels and periods of rest, they do not accurately differentiate between resting and actual sleep. Additionally, total activity was calculated as an average over extended periods of time, potentially overlooking specific changes that may occur at different times of the day. To further examine the relationship between epilepsy and sleep disturbances in dogs, future studies should incorporate polysomnography, the gold‐standard technique to measure sleep architecture [29, 30]. In our study, owners were asked about their perceptions of changes in their dogs' sleep patterns after seizures. More than half of the respondents reported that their dogs slept more than usual after a seizure. However, some owners noted that their dogs experienced difficulty falling asleep or exhibited altered sleep characteristics, such as sleeping with their eyes open or having variable sleep patterns.

Interestingly, although we found that dogs with epilepsy appeared to have poorer sleep quality, their owners perceived increased sleepiness in these dogs immediately after a seizure. A recent study confirmed that lethargy and sleepiness are reported postictal signs in dogs, but not the most common, and not signs with high impact on the dog's or owner's quality of life [31]. Most owners noted that after a seizure, their dogs tend to be disoriented, compulsively walk, and show signs of ataxia. When owners were asked what alleviates the impact of postictal signs, most responded that rest, a quiet environment, and darkness were helpful. These findings suggest that owners might be trying to create a more favorable environment for sleep after a seizure, increasing the chances of the dog falling asleep after the most common postictal signs have subsided.

Advancing age [32] and cognitive decline [22] can impair sleep. Therefore, when the SNoRE questionnaire was validated, construct validity was assessed in a cohort of senior dogs with and without cognitive dysfunction by correlating the scores with a validated canine dementia scale (CADES). The median score obtained on the SNoRE questionnaire in the epileptic dogs of our study was higher than in the cohort of senior dogs from our previous study [25] (12 [range: 6–33] vs. 9 [range: 6–30], respectively). Our findings suggest that the relationship between epilepsy and sleep in dogs could be even more clinically relevant than the relationship between aging and cognitive impairment.

Dogs with epilepsy have been reported to experience cognitive impairments, such as working memory deficits [33] and early onset of cognitive dysfunction [34]. This association has also been observed in humans and rodents and is thought to result from epileptic activity (including both seizures and interictal spikes) as well as from the structural and functional abnormalities seen in epilepsy [35, 36]. Moreover, interictal discharges and seizures frequently occur during NREM sleep, a stage essential for memory consolidation [37]. We believe that sleep disturbances in epileptic patients might contribute to cognitive deficits, and additional studies should evaluate the relationship between sleep loss and performance in cognitive testing.

The lack of association between SNoRE scores and seizure frequency, the presence of cluster seizures, or ASMs was unexpected, because in humans, sleep instability has been associated with higher seizure frequency and the presence of cluster seizures [38, 39]. Additionally, as previously mentioned, ASMs have been shown to influence sleep [15, 18, 24]. Specifically, in dogs, two different studies found that dogs treated with KBr had lower sleep scores, indicating a worse sleep quality or time [24, 40]. In our study, the median score of factor 1 was higher in dogs on KBr in comparison with dogs not receiving KBr (10 vs. 8, respectively); however, differences were not significant. One potential explanation for these findings is that our analyses were underpowered, which could limit the detection of subtle differences. This is particularly true for detecting the effects of ASMs, given the heterogeneity of our population of dogs and their treatments. Some of the dogs also were treated with supplements that can induce sleep such as melatonin [41], CBD [42, 43], and MCT oil [44]. A larger sample size within each category and less heterogeneous treatment groups may be necessary to identify associations that were not evident in this study.

The association between sleep and epilepsy in dogs should prompt further research on potential interventions that could improve both seizure frequency and sleep quality. In this regard, a recent study evaluated increased exercise as a non‐pharmacological intervention in epileptic dogs. Although exercise improved sleep quality, it significantly worsened seizure frequency. Other potential interventions could include administering sleep‐promoting drugs or supplements. In this context, melatonin, a sleep‐promoting indole hormone, has been shown to improve sleep in children with epilepsy [45, 46], but its effects on seizure control vary among studies. Some report no change in seizure frequency [45], whereas others found improvements in seizure frequency [47], severity [48], and a decrease in the electroencephalographic interictal activity [46]. A pro‐convulsant effect also has been reported [49]. One study compared serum melatonin concentrations in dogs with epilepsy to healthy controls and found no significant differences [50]. However, most samples had undetectable melatonin concentrations (< 0.5 pg/mL) based on radioimmunoassay. Therefore, a difference in melatonin concentrations between epileptic and healthy dogs might still exist but could fall below the assay's detectable range. Alternatively, a different detection method could provide more insights. To our knowledge, the effect of melatonin administration on epileptic dogs has not yet been evaluated.

Our study had several limitations and was intended as an exploratory study to encourage future larger multicenter studies. First, although the SNoRE questionnaire has been validated using more objective tools such as polysomnography and activity monitors, it still relies on owner‐reported data, which can be influenced by recall bias and subjective interpretation of their dogs' sleep. Second, the study did not account for the effects of other medical conditions or medications on sleep, and data on the type of seizures (generalized vs. focal) as well as comorbidities in the epileptic dogs were not gathered. Additionally, we did not measure serum concentrations of ASMs, which could influence the results. Although we used matching controls based on age, breed, and sex, we did not include confounding variables that could influence sleep such as body condition score [51] or pre‐sleep activity [52] in the statistical analysis. Finally, the small sample size and lack of more than one matched control per epileptic dog, along with variations in seizure frequency and antiseizure medications, may limit the generalizability of our findings.

In conclusion, we showed that idiopathic epilepsy is associated with a worsening of sleep quality in dogs, highlighting the importance of monitoring and addressing sleep disturbances in these patients. Future multicenter studies with larger and more diverse populations, adjusted for potential confounding variables, and using objective sleep measurements such as polysomnography, are necessary to determine the generalizability of our findings to a broader population of dogs and to better understand the bidirectional interaction between sleep and epilepsy.

## Disclosure

Authors declare no off‐label use of antimicrobials.

## Ethics Statement

Approved by the North Carolina State University Institutional Animal Care and Use Committee under protocol number 23–334. Authors declare human ethics approval was not needed.

## Conflicts of Interest

The authors declare no conflicts of interest.

## Supporting information


**File S1.** Supporting Information.

**File S2.** Supporting Information.

## References

[1] L. Kearsley‐Fleet , D. G. O'Neill , H. A. Volk , et al., “Prevalence and Risk Factors for Canine Epilepsy of Unknown Origin in the UK,” Veterinary Record 172 (2013): 338. 23300065 10.1136/vr.101133

[2] A. Erlen , H. Potschka , H. A. Volk , C. Sauter‐Louis , and D. G. O'Neill , “Seizure Occurrence in Dogs Under Primary Veterinary Care in the UK: Prevalence and Risk Factors,” Journal of Veterinary Internal Medicine 32 (2018): 1665–1676. 30216557 10.1111/jvim.15290 PMC6189390

[3] L. Heske , A. Nødtvedt , K. H. Jäderlund , M. Berendt , and A. Egenvall , “A Cohort Study of Epilepsy Among 665,000 Insured Dogs: Incidence, Mortality and Survival After Diagnosis,” Veterinary Journal 202 (2014): 471–476. 25457266 10.1016/j.tvjl.2014.09.023

[4] M. Berendt , H. Gredal , L. G. Pedersen , L. Alban , and J. Alving , “A Cross‐Sectional Study of Epilepsy in Danish Labrador Retrievers: Prevalence and Selected Risk Factors,” Journal of Veterinary Internal Medicine 16 (2002): 262–268. 12041655 10.1892/0891-6640(2002)016<0262:acsoei>2.3.co;2

[5] M. Berendt , C. H. Gulløv , S. L. K. Christensen , et al., “Prevalence and Characteristics of Epilepsy in the Belgian Shepherd Variants Groenendael and Tervueren Born in Denmark 1995‐2004,” Acta Veterinaria Scandinavica 50 (2008): 51. 19102738 10.1186/1751-0147-50-51 PMC2633289

[6] C. H. Gulløv , N. Toft , M. M. N. Baadsager , and M. Berendt , “Epilepsy in the Petit Basset Griffon Vendeen: Prevalence, Semiology, and Clinical Phenotype,” Journal of Veterinary Internal Medicine 25 (2011): 1372–1378. 22092630 10.1111/j.1939-1676.2011.00791.x

[7] V. I. Hülsmeyer , A. Fischer , P. J. J. Mandigers , et al., “International Veterinary Epilepsy Task Force's Current Understanding of Idiopathic Epilepsy of Genetic or Suspected Genetic Origin in Purebred Dogs,” BMC Veterinary Research 11 (2015): 175. 26316206 10.1186/s12917-015-0463-0 PMC4552344

[8] V. Kokkinos , A. M. Koupparis , M. Koutroumanidis , and G. K. Kostopoulos , “Editorial: Brain Mechanisms Linking Sleep and Epilepsy,” Frontiers in Human Neuroscience 16 (2022): 922372. 35620153 10.3389/fnhum.2022.922372 PMC9128402

[9] D. Minecan , A. Natarajan , M. Marzec , and B. Malow , “Relationship of Epileptic Seizures to Sleep Stage and Sleep Depth,” Sleep 25 (2002): 899–904. 12489898

[10] B. A. Malow , X. Lin , R. Kushwaha , and M. S. Aldrich , “Interictal Spiking Increases With Sleep Depth in Temporal Lobe Epilepsy,” Epilepsia 39 (1998): 1309–1316. 9860066 10.1111/j.1528-1157.1998.tb01329.x

[11] X. Yuan and M. Sun , “The Value of Rapid Eye Movement Sleep in the Localization of Epileptogenic Foci for Patients With Focal Epilepsy,” Seizure 81 (2020): 192–197. 32854037 10.1016/j.seizure.2020.06.009

[12] R. Manni and M. Terzaghi , “Comorbidity Between Epilepsy and Sleep Disorders,” Epilepsy Research 90 (2010): 171–177. 20570109 10.1016/j.eplepsyres.2010.05.006

[13] P. Krishnan , S. Sinha , A. B. Taly , C. T. Ramachandraiah , S. Rao , and P. Satishchandra , “Sleep Disturbances in Juvenile Myoclonic Epilepsy: A Sleep Questionnaire‐Based Study,” Epilepsy & Behavior 23 (2012): 305–309. 22366052 10.1016/j.yebeh.2011.12.018

[14] M. M. Frucht , M. Quigg , C. Schwaner , and N. B. Fountain , “Distribution of Seizure Precipitants Among Epilepsy Syndromes,” Epilepsia 41 (2000): 1534–1539. 11114210 10.1111/j.1499-1654.2000.001534.x

[15] B. M. S. Carvalho , J. Chaves , and A. M. da Silva , “Effects of Antiepileptic Drugs on Sleep Architecture Parameters in Adults,” Sleep Science 15 (2022): 224–244. 10.5935/1984-0063.20220045 PMC9210559 35755913

[16] B. Legros and C. W. Bazil , “Effects of Antiepileptic Drugs on Sleep Architecture: A Pilot Study,” Sleep Medicine 4 (2003): 51–55. 14592360 10.1016/s1389-9457(02)00217-4

[17] A. Cicolin , U. Magliola , A. Giordano , A. Terreni , C. Bucca , and R. Mutani , “Effects of Levetiracetam on Nocturnal Sleep and Daytime Vigilance in Healthy Volunteers,” Epilepsia 47 (2006): 82–85. 10.1111/j.1528-1167.2006.00376.x 16417535

[18] C. Bell , H. Vanderlinden , R. Hiersemenzel , C. Otoul , D. Nutt , and S. Wilson , “The Effects of Levetiracetam on Objective and Subjective Sleep Parameters in Healthy Volunteers and Patients With Partial Epilepsy,” Journal of Sleep Research 11 (2002): 255–263. 12220322 10.1046/j.1365-2869.2002.00301.x

[19] J. Y. Zhou , X. D. Tang , L. L. Huang , Z. Q. Zhong , F. Lei , and D. Zhou , “The Acute Effects of Levetiracetam on Nocturnal Sleep and Daytime Sleepiness in Patients With Partial Epilepsy,” Journal of Clinical Neuroscience 19 (2012): 956–960. 22595355 10.1016/j.jocn.2011.09.032

[20] C. W. Bazil , J. Battista , and R. C. Basner , “Effects of Levetiracetam on Sleep in Normal Volunteers,” Epilepsy & Behavior 7 (2005): 539–542. 16165400 10.1016/j.yebeh.2005.08.001

[21] A. Mondino , L. Delucchi , A. Moeser , S. Cerdá‐González , and G. Vanini , “Sleep Disorders in Dogs: A Pathophysiological and Clinical Review,” Topics in Companion Animal Medicine 43 (2021): 100516. 33556640 10.1016/j.tcam.2021.100516

[22] A. Mondino , M. Catanzariti , D. M. Mateos , et al., “Sleep and Cognition in Aging Dogs. A Polysomnographic Study,” Frontiers in Veterinary Science 10 (2023): 1151266. 37187924 10.3389/fvets.2023.1151266 PMC10175583

[23] T. A. Schubert , R. M. Chidester , and C. L. Chrisman , “Clinical Characteristics, Management and Long‐Term Outcome of Suspected Rapid Eye Movement Sleep Behaviour Disorder in 14 Dogs,” Journal of Small Animal Practice 52 (2011): 93–100. 21265848 10.1111/j.1748-5827.2010.01026.x

[24] M. Barry , S. Cameron , S. Kent , H. Barnes‐Heller , and K. Grady , “Daytime and Nocturnal Activity in Treated Dogs With Idiopathic Epilepsy Compared to Matched Unaffected Controls,” Journal of Veterinary Internal Medicine 35 (2021): 1826–1833. 34223667 10.1111/jvim.16205 PMC8295678

[25] A. Mondino , C. Ludwig , C. Menchaca , et al., “Development and Validation of a Sleep Questionnaire, SNoRE 3.0, to Evaluate Sleep in Companion Dogs,” Scientific Reports 13 (2023): 13340. 37587172 10.1038/s41598-023-40048-1 PMC10432410

[26] L. De Risio , S. Bhatti , K. Muñana , et al., “International Veterinary Epilepsy Task Force Consensus Proposal: Diagnostic Approach to Epilepsy in Dogs,” BMC Veterinary Research 11 (2015): 148. 26316175 10.1186/s12917-015-0462-1 PMC4552251

[27] J. Lehner , J. S. Frueh , and A. N. Datta , “Sleep Quality and Architecture in Idiopathic Generalized Epilepsy: A Systematic Review and Meta‐Analysis,” Sleep Medicine Reviews 65 (2022): 101689. 36037570 10.1016/j.smrv.2022.101689

[28] R. Manni , M. Terzaghi , and E. Zambrelli , “REM Sleep Behaviour Disorder in Elderly Subjects With Epilepsy: Frequency and Clinical Aspects of the Comorbidity,” Epilepsy Research 77 (2007): 128–133. 17980556 10.1016/j.eplepsyres.2007.09.007

[29] A. Kis , S. Szakadát , E. Kovács , et al., “Development of a Non‐invasive Polysomnography Technique for Dogs (Canis familiaris),” Physiology & Behavior 130 (2014): 149–156. 24726397 10.1016/j.physbeh.2014.04.004

[30] P. Torterolo , J. Gonzalez , S. Castro‐Zaballa , et al., “Polysomnography in Humans and Animal Models,” in Methodological Approaches for Sleep and Vigilance Research, ed. E. Murillo‐Rodríguez (Elsevier, 2021), 17–32.

[31] C. Kähn , N. Meyerhoff , S. Meller , J. N. Nessler , H. A. Volk , and M. Charalambous , “The Postictal Phase in Canine Idiopathic Epilepsy: Semiology, Management, and Impact on the Quality of Life From the Owners' Perspective,” Animals 14 (2024): 103. 10.3390/ani14010103 PMC10778387 38200833

[32] T. Takeuchi and E. Harada , “Age‐Related Changes in Sleep‐Wake Rhythm in Dog,” Behavioural Brain Research 136 (2002): 193–199. 12385805 10.1016/s0166-4328(02)00123-7

[33] J. Winter , R. M. A. Packer , and H. A. Volk , “Preliminary Assessment of Cognitive Impairments in Canine Idiopathic Epilepsy,” Veterinary Record 182 (2018): 633. 29700175 10.1136/vr.104603

[34] R. M. A. Packer , P. D. McGreevy , H. E. Salvin , et al., “Cognitive Dysfunction in Naturally Occurring Canine Idiopathic Epilepsy,” PLoS One 13 (2018): e0192182. 29420639 10.1371/journal.pone.0192182 PMC5805257

[35] G. L. Holmes , “Cognitive Impairment in Epilepsy: The Role of Network Abnormalities,” Epileptic Disorders 17 (2015): 101–116. 25905906 10.1684/epd.2015.0739 PMC5410366

[36] R. A. B. Badawy , K. A. Johnson , M. J. Cook , and A. S. Harvey , “A Mechanistic Appraisal of Cognitive Dysfunction in Epilepsy,” Neuroscience and Biobehavioral Reviews 36 (2012): 1885–1896. 22617705 10.1016/j.neubiorev.2012.05.002

[37] S. Ackermann and B. Rasch , “Differential Effects of Non‐REM and REM Sleep on Memory Consolidation?,” Current Neurology and Neuroscience Reports 14 (2014): 430. 24395522 10.1007/s11910-013-0430-8

[38] R. Manni , E. Zambrelli , R. Bellazzi , and M. Terzaghi , “The Relationship Between Focal Seizures and Sleep: An Analysis of the Cyclic Alternating Pattern,” Epilepsy Research 67 (2005): 73–80. 16185848 10.1016/j.eplepsyres.2005.08.008

[39] P. Kotagal and N. Yardi , “The Relationship Between Sleep and Epilepsy,” Seminars in Pediatric Neurology 15 (2008): 42–49. 18555190 10.1016/j.spen.2008.03.007

[40] K. Grady , S. Cameron , S. P. Kent , H. Barnes Heller , and M. M. Barry , “Effect of an Intervention of Exercise on Sleep and Seizure Frequency in Idiopathic Epileptic Dogs,” Journal of Small Animal Practice 64 (2023): 59–68. 36368312 10.1111/jsap.13568 PMC10099787

[41] A. Brzezinski , M. G. Vangel , R. J. Wurtman , et al., “Effects of Exogenous Melatonin on Sleep: A Meta‐Analysis,” Sleep Medicine Reviews 9 (2005): 41–50. 15649737 10.1016/j.smrv.2004.06.004

[42] E. B. Russo , G. W. Guy , and P. J. Robson , “Cannabis, Pain, and Sleep: Lessons From Therapeutic Clinical Trials of Sativex®, a Cannabis‐Based Medicine,” Chemistry & Biodiversity 4 (2007): 1729–1743. 17712817 10.1002/cbdv.200790150

[43] A. Mondino , M. Cavelli , J. González , et al., “Effects of Cannabis Consumption on Sleep,” in Cannabinoids and Sleep: Molecular, Functional and Clinical Advances, ed. J. M. Monti , S. R. Pandi‐Perumal , and E. Murillo‐Rodríguez (Springer, 2021), 147–162. 10.1007/978-3-030-61663-2_11 33537943

[44] F. Telliez , V. Bach , A. Leke , K. Chardon , and J. P. Libert , “Feeding Behavior in Neonates Whose Diet Contained Medium‐Chain Triacylglycerols: Short‐Term Effects on Thermoregulation and Sleep,” American Journal of Clinical Nutrition 76 (2002): 1091–1095. 12399283 10.1093/ajcn/76.5.1091

[45] S. V. Jain , P. S. Horn , N. Simakajornboon , et al., “Melatonin Improves Sleep in Children With Epilepsy: A Randomized, Double‐Blind, Crossover Study,” Sleep Medicine 16 (2015): 637–644. 25862116 10.1016/j.sleep.2015.01.005 PMC4425994

[46] G. B. Dell'Isola , G. Tascini , V. Vinti , et al., “Effect of Melatonin on Sleep Quality and EEG Features in Childhood Epilepsy: A Possible Non‐conventional Treatment,” Frontiers in Neurology 14 (2023): 1243917. 37780697 10.3389/fneur.2023.1243917 PMC10538564

[47] N. Peled , Z. Shorer , E. Peled , and G. Pillar , “Melatonin Effect on Seizures in Children With Severe Neurologic Deficit Disorders,” Epilepsia 42 (2001): 1208–1210. 11580772 10.1046/j.1528-1157.2001.28100.x

[48] H. A. Elkhayat , S. M. Hassanein , H. Y. Tomoum , I. A. Abd‐Elhamid , T. Asaad , and A. S. Elwakkad , “Melatonin and Sleep‐Related Problems in Children With Intractable Epilepsy,” Pediatric Neurology 42 (2010): 249–254. 20304327 10.1016/j.pediatrneurol.2009.11.002

[49] S. H. Sheldon , “Pro‐Convulsant Effects of Oral Melatonin in Neurologically Disabled Children,” Lancet 351 (1998): 1254. 9643754 10.1016/S0140-6736(05)79321-1

[50] S. A. Thomovsky , A. V. Chen , D. M. Deavila , and A. M. Kiszonas , “Serum Melatonin Values in Normal Dogs and Dogs With Seizures,” Journal of the American Animal Hospital Association 55 (2019): 78–82. 30653360 10.5326/JAAHA-MS-6669

[51] I. Niinikoski , S. L. Himanen , M. Tenhunen , M. Aromaa , L. Lilja‐Maula , and M. M. Rajamäki , “Evaluation of Risk Factors for Sleep‐Disordered Breathing in Dogs,” Journal of Veterinary Internal Medicine 38 (2024): 1135–1145. 38358051 10.1111/jvim.17019 PMC10937515

[52] N. Bunford , V. Reicher , A. Kis , et al., “Differences in Pre‐Sleep Activity and Sleep Location Are Associated With Variability in Daytime/Nighttime Sleep Electrophysiology in the Domestic Dog,” Scientific Reports 8 (2018): 7109. 29740040 10.1038/s41598-018-25546-x PMC5940857

