# Supplementary material for: An Exploratory Study on the Relationship Between Idiopathic Epilepsy and Sleep in Dogs
Source: J Vet Intern Med. 2025 Mar 15;39(2):e70026. doi: 10.1111/jvim.70026 (PMC11910028; doi:10.1111/jvim.70026)
Supplement: Supplementary file 1 — File S1. Supporting Information. [file JVIM-39-e70026-s001.pdf]

Please provide your name (first and last):

Email address:

Dog's name:

Dog's date of birth (exact or estimated):

Dog's breed (If mixed breed, please also include predominant breed if known):

Dogs' sex:

- ○ Intact Male
- ○ Neutered Male

○ Intact Female
○ Spayed Female

Epilepsy History

How old was your dog at the time of the first seizure?

Provide the date of the first seizure if known (MM/DD/YYYY):

What testing was performed to diagnose your dog's epilepsy?

- ☐ Bloodwork (CBC, Chemistry profile)
- ☐ Bile acid testing
- ☐ Magnetic Resonance Imaging (MRI)
- ☐ Computed Tomography (CT Scan)
- ☐ Cerebral Spinal Fluid (CSF) Analysis
- ☐ Other

What time of day do your dog’s seizures tend to occur?

- ○ Morning
- ○ Afternoon
- ○ Evening
- ○ Overnight
- ○ No specific time of the day

Is your dog's behavior normal between seizures?

- ○ Yes
- ○ No

If you answered NO, to the above question, which of the following abnormal behaviors does your dog display between seizures (check all that apply):

- ☐ Attention seeking
- ☐ Fear
- ☐ Aggression
- ☐ Anxiety
- ☐ Circling/pacing
- ☐ Separation-related behaviors
- ☐ Decreased trainability
- ☐ Touch/sound sensitivity
- ☐ Hyperexcitability
- ☐ Failure to recognize familiar people
- ☐ Reduced interest in activities

☐ ☐ Other (please describe)

Under which of the following circumstances does your dog's seizure activity usually occur? (More than 1 answer can be selected):

☐ At rest
☐ During sleep
☐ During exercise
☐ During stress/excitement
☐ Soon after a meal
☐ ☐ Other (Please describe)

On average, how often has your dog had a seizure episode in the last 12 months?

○ Daily
○ Weekly
○ Monthly
○ Every 2-6 months
○ Yearly

How long ago was your dog's last seizure event? (Please indicate the date of seizure event if known)

Has your dog ever had more than one seizure within 24 hours?

- ○ Yes
- ○ No

Which statement fits best with your dog's most common seizure presentation?

- ○ Isolated seizures only (not more frequent than one a day)
- ○ Cluster seizures (more than one seizure a day)
- ○ Both isolated seizures and cluster seizures on separate occasions

Does your dog display abnormal behavior after a seizure?

- ○ Yes
- ○ No

If you answered YES, to the above question, which of the following abnormal signs does your dog display immediately **AFTER** having a seizure (check all that apply):

- ☐ Attention seeking
- ☐ Fearful
- ☐ Aggressive

☐ Sleepy/lethargic
☐ Wobbly/clumsy
☐ Blind
☐ Disoriented
☐ Vocalization
☐ Weak on all four legs
☐ Weak on back legs only
☐ Weak on one side (one front leg and one back leg)
☐ Hungry
☐ Thirsty
☐ Deafness
☐ [ ] Other: Please describe:

Does your dog show changes in their sleep or wakefulness after a seizure? (You can select more than one answer)

☐ Yes, they have difficulty falling asleep
☐ Yes, they sleep more than usual
☐ [ ] Yes, other (please describe)
☐ No

Which of the following anti-seizure medication is your dog currently managed on?

☐ Phenobarbital

☐ Potasssium bromide
☐ Zonisamide
☐ Levetiracetam (Keppra)
☐ Pregabalin
☐ Benzodiazepines (diazepam, midazolam, or clorazepate)
☐ [ ] Other (please describe)
☐ None

Which of the following anti-seizure medications has been used to treat seizures in your dog in the past?

☐ Phenobarbital
☐ Potassium Bromide
☐ Zonisamide
☐ Levetiracetam (Keppra)
☐ Pregabalin
☐ Benzodiazepines (diazepam, midazolam or clorazepate)
☐ [ ] Other (please describe)
☐ None

If your pet is receiving any other medications or supplements, please list below:

[ ]

Sleep Questionnaire

Over the last 7 days, what has been your dog's ability to sleep at bedtime? Choose the number that best describes your dog's ability to sleep (1-Falls asleep easily at bedtime; 10-Takes much longer than usual to fall asleep):

1	2	3	4	5	6	7	8	9	10
○	○	○	○	○	○	○	○	○	○

Over the last 7 days, what has been your dog's ability to sleep continuously during the night? Choose the number that best describes your dog's ability to sleep (1-Never gets up/paces; 10-Constantly getting up/pacing):

1	2	3	4	5	6	7	8	9	10
○	○	○	○	○	○	○	○	○	○

Choose the number that best describes your dog's sleep over the last 7 days: (1-Never needs to eliminate during the night; 10-Needs to eliminate several times every night):

1	2	3	4	5	6	7	8	9	10
○	○	○	○	○	○	○	○	○	○

Choose the number that best describes your dog's breathing while sleeping over the last 7 days:
(1-Never pauses breathing; 10-Has several breathing interruptions)

1	2	3	4	5	6	7	8	9	10
○	○	○	○	○	○	○	○	○	○

Choose the one number that best describes how much their own vocalizations (while dreaming) affected your dog's sleep over the last 7 days. (1-Vocalizations (whimpering, barking) never wake them up; 10-Vocalizations (whimpering, barking) are so loud or frequent that they constantly wake them up):

1	2	3	4	5	6	7	8	9	10
○	○	○	○	○	○	○	○	○	○

Choose the number that best describes how much twitching affected your dog's sleep over the last 7 days:
(1-Twitching never wakes them up; 10-Twitches are so frequent and/or severe they constantly wake them up):

1	2	3	4	5	6	7	8	9	10
○	○	○	○	○	○	○	○	○	○

Is there anything more that you would like to add about your pet and sleep patterns, that we did not address in this survey or that you would like to add? Please comment below:
