# Supplementary material for: An Exploratory Study on the Relationship Between Idiopathic Epilepsy and Sleep in Dogs
Source: J Vet Intern Med. 2025 Mar 15;39(2):e70026. doi: 10.1111/jvim.70026 (PMC11910028; doi:10.1111/jvim.70026)
Supplement: Supplementary file 2 — File S2. Supporting Information. [file JVIM-39-e70026-s002.pdf]

Please provide your first name:

Please provide your last name:

Email address:

Dog's name:

Dog's date of birth (exact or estimated):

Dog's breed (If mixed breed, please also include predominant

breed if known):

Dogs' sex:

- ○ Intact Male
- ○ Neutered Male
- ○ Intact Female
- ○ Spayed Female

Medical history

Has your dog ever been diagnosed with any disease or medical condition?

- ○ Yes
- ○ No

Please list the diagnoses below

Are any of these medical conditions currently affecting your dog?

○ Yes

○ No

Please list your dog's current medical conditions

Is your dog currently taking any medications or supplements?

○ Yes

○ No

Please list the medications or supplements below

Has your dog ever experienced a seizure?

○ Yes

○ No

Please add any additional information about your dog's health history that you believe may be important for us to know

Sleep Questionnaire - sleep section corrected

Over the last 7 days, what has been your dog's ability to sleep at bedtime? Choose the number that best describes your dog's ability to sleep
(1-Falls asleep easily at bedtime; 10-Takes much longer than usual to fall asleep):

1 ○ 2 ○ 3 ○ 4 ○ 5 ○ 6 ○ 7 ○ 8 ○ 9 ○ 10 ○

Over the last 7 days, what has been your dog's ability to sleep continuously during the night? Choose the number that best describes your dog's ability to sleep (1-Never gets up/paces; 10-Constantly getting up/pacing):

1 ○ 2 ○ 3 ○ 4 ○ 5 ○ 6 ○ 7 ○ 8 ○ 9 ○ 10 ○

Choose the number that best describes your dog's sleep over the last 7 days:
(1-Never needs to eliminate during the night; 10-Needs to eliminate several times every night):

1 ○ 2 ○ 3 ○ 4 ○ 5 ○ 6 ○ 7 ○ 8 ○ 9 ○ 10 ○

Choose the number that best describes your dog's breathing while sleeping over the last 7 days:
(1-Never pauses breathing; 10-Has several breathing interruptions)

1	2	3	4	5	6	7	8	9	10
○	○	○	○	○	○	○	○	○	○

Choose the one number that best describes how much their own vocalizations (while dreaming) affected your dog's sleep over the last 7 days. (1-Vocalizations (whimpering, barking) never wake them up; 10-Vocalizations (whimpering, barking) are so loud or frequent that they constantly wake them up):

1	2	3	4	5	6	7	8	9	10
○	○	○	○	○	○	○	○	○	○

Choose the number that best describes how much twitching affected your dog's sleep over the last 7 days:
(1-Twitching never wakes them up; 10-Twitches are so frequent and/or severe they constantly wake them up):

1	2	3	4	5	6	7	8	9	10
○	○	○	○	○	○	○	○	○	○

Is there anything more that you would like to add about your pet and sleep patterns, that we did not address in this survey or that you would like to add? Please comment below:
